# Survival after breast-conserving surgery and radiotherapy *versus* mastectomy: propensity score analyses within a randomized anaesthesiology trial

**DOI:** 10.1093/bjs/znag036

**Published:** 2026-04-07

**Authors:** Charlotta Wadsten, Anders Berglund, Emma Söderberg, Greger Nilsson, Leif Bergkvist, Mats Enlund, Fredrik Wärnberg

**Affiliations:** Department of Surgery, Sundsvall Hospital, Sundsvall, Sweden; Department of Diagnostics and Intervention, Surgery, Umeå University, Umeå, Sweden; Department of Statistics, Epistat AB, Uppsala, Sweden; Department of Surgery, Sundsvall Hospital, Sundsvall, Sweden; Department of Diagnostics and Intervention, Surgery, Umeå University, Umeå, Sweden; Department of Immunology, Genetics, and Pathology, Uppsala University, Uppsala, Sweden; Department of Oncology, Gävle Hospital, Gävle, Sweden; Department of Oncology, Visby Hospital, Visby, Sweden; Centre for Clinical Research, Uppsala University and Region Vastmanland, Vastmanland Hospital, Västerås, Sweden; Centre for Clinical Research, Uppsala University and Region Vastmanland, Vastmanland Hospital, Västerås, Sweden; Department of Surgical Sciences, Division of Anaesthesiology and Intensive Care, Uppsala University, Uppsala, Sweden; ESAIC Onco Anaestehesiology Group, EuroPeriscope; Department of Surgery, Sahlgrenska University Hospital, Gothenburg, Sweden; Institution of Clinical Sciences, Sahlgrenska Academy at Gothenburg University, Gothenburg, Sweden

## Abstract

**Background:**

Large randomized studies show equivalent survival after breast-conserving surgery (BCS) + adjuvant radiotherapy (RT) and mastectomy. In contrast, more recent observational studies suggest BCS + RT to be superior, but it is questionable whether patients in these treatment arms are comparable. Here, overall survival (OS) and breast cancer-specific survival (BCSS) after BCS + RT and mastectomy are compared within a randomized trial comparing intravenous and inhalation anaesthesia during breast cancer surgery.

**Methods:**

The patient cohort was recruited from the randomized CAN-study. Patients with tumours >30 mm, patients with tumours <10 mm, and patients who underwent BCS without RT were excluded. OS and BCSS were estimated using multivariable Cox regression analyses and three different propensity score models.

**Results:**

The final study cohort included 830 women, of whom 601 underwent BCS + RT (median age 64 years) and 229 underwent mastectomy (median age 68 years). Women who underwent mastectomy had more co-morbidities and more unfavourable tumour characteristics. Mastectomy was associated with significantly less favourable OS in unadjusted, adjusted, and two of the three propensity score analyses. BCSS was inferior in the mastectomy group in the unadjusted analysis, with an HR of 2.27 (95% c.i. 1.20 to 4.30). In the adjusted and three propensity score analyses, BCSS was equal in the treatment groups, with an adjusted HR of 1.02 (95% c.i. 0.43 to 2.42).

**Conclusion:**

In this study, which was designed to approximate a randomized trial as closely as possible, no significant difference in BCSS was observed between BCS + RT and mastectomy. Differences in OS likely reflect occult selection bias of patients with higher co-morbidity burden to mastectomy.

## Teaser

Large randomized trials show equivalent survival after breast-conserving surgery plus radiotherapy (BCS + RT) and mastectomy, though observational studies have suggested a benefit for BCS + RT. In this analysis of 830 women from a randomized trial cohort, mastectomy was associated with worse overall survival, but breast cancer-specific survival (BCSS) did not differ between BCS + RT and mastectomy after adjustment and propensity score matching/weighting. These findings support comparable BCSS between the two surgical approaches when patient characteristics are carefully balanced.

## Introduction

Since the early 1990s, breast conservation has been recommended as the primary surgical choice for early breast cancer. This is based on results from several randomized studies, comparing breast-conserving surgery (BCS) with adjuvant radiotherapy (RT) and mastectomy^1,2^. Overall survival (OS) and breast cancer-specific survival (BCSS) were shown to be similar after 20 years, but with a higher risk of local recurrence after BCS^3,4^. Presenting BCS + RT as the primary surgical choice for women when BCS is possible is not controversial, but, still, mastectomy is often opted for instead of BCS+RT. This can be due to slow adaptation to BCS among surgeons, but can sometimes also depend on patient preferences. Today, BCS + RT is recommended, as it is a less extensive procedure, with a lower risk of complications^5^. Notwithstanding, some women opt for mastectomy for different reasons, one being a concern that the cancer will recur.

During recent years, a formidable surge of reports has been published, showing that survival after BCS + RT is better than survival after mastectomy^6–14^. These studies are mainly register based or observational retrospective studies, and, although adjustments are done, a residual risk of confounding remains. This raises the question of whether patients in the treatment arms of such studies are really comparable. As an example, the decision regarding surgical method is based on preoperative tumour characteristics, whereas adjustments most often use postoperative data. The reason for a mastectomy is seldom reported in the medical records or registries, which may lead to differences that might be impossible to adjust for. To try to mimic randomized trials, propensity score-matched (PSM) data have been published on survival after BCS + RT and mastectomy with diverging results^15–18^ and with the same remaining question, are factors that impact the choice of surgery and that are related to prognosis missed and the data confounded?

While the proportion of mastectomies is steadily falling in Sweden, there are reports on an increasing frequency of mastectomies in other countries^19^. The challenge is to communicate the current scientific knowledge to patients so that informed and shared treatment decisions are made. The present study is a comparison of OS and BCSS after BCS + RT and mastectomy within a randomized trial comparing intravenous and inhalation anaesthesia during breast cancer surgery. The analyses include crude and adjusted analyses, as well as three different propensity score analyses.

## Methods

### Study population and design

The patient cohort was recruited from the randomized CAN-study^20^, comparing the impact of propofol and sevoflurane on breast cancer survival in Sweden and China. A 5-year follow-up was published in 2023 and showed that the type of anaesthesia had no impact on survival. Patients of both sexes, aged ≥18 years, who were scheduled for elective primary breast cancer surgery with curative intent under general anaesthesia, with or without local anaesthesia, were included in the CAN-study. The study cohort was recruited between 2013 and 2017. Patients scheduled for emergency or palliative surgery, with carcinoma *in situ*, with the presence of any contraindication (according to the valid Summary of Product Characteristics for each substance), or with lack of suitability for any reason, as judged by the local investigator, for example communicative disturbances (language or intellectual), were not included in the study.

Patient and tumour characteristics were collected from the CAN-study database and complemented with data from the Swedish National Breast Cancer Quality Register (NKBC) regarding tumour size (in mm) and human epidermal growth factor receptor 2 (HER2) status. Data on time of death and cause of death were collected from the Swedish National Cause of Death Registry, which has high validity regarding the reporting of breast cancer deaths^21^.

**Fig. 1:**
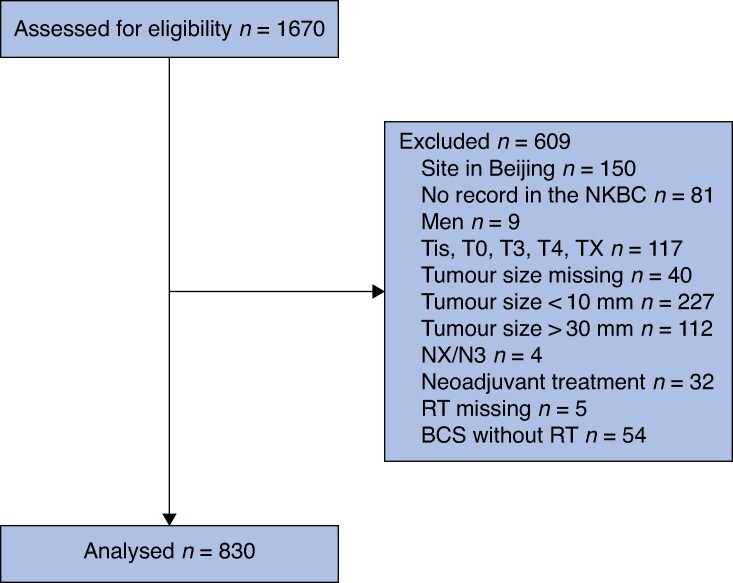
Flow chart of eligible patients NKBC, Swedish National Breast Cancer Quality Register; RT, radiotherapy; BCS, breast-conserving surgery.

Of the 1670 patients included in the CAN-study, the following were excluded from the present analysis: patients from China due to lack of data on tumour characteristics, male patients, patients who underwent BCS without RT, patients who received neoadjuvant treatment and patients with some missing data (*Fig. 1*). Furthermore, patients with tumours >30 mm or <10 mm were excluded. These size cut-offs were used with the intention of excluding patients where BCS might not have been an option and patients with very small tumours who underwent mastectomy for unknown reasons that could not be adjusted or matched for, such as hereditary risk or RT contraindications. In the different analyses, slightly different cohorts were used, depending on available data and matching. For the crude analyses, all patients were included. In the adjusted analyses, all variables in *Table 1* (patient characteristics) and *Table 2* (tumour characteristics and adjuvant oncological treatment) were included.

**Table 1:** Patient characteristics for the BCS + RT and mastectomy groups

	BCS + RT (*n* = 601)	Mastectomy (*n* = 229)	SMD	Missing
Age (years), median (i.q.r.)	64.0 (53.0–70.0)	68.0 (53.0–76.0)	0.219	–
**Age group (years)**				
27–49	98 (16)	46 (20)	0.548	–
50–59	125 (20.8)	33 (14)		
60–69	210 (34.9)	54 (24)		
70–79	146 (24.3)	53 (23)		
≥80	22 (4)	43 (19)		
BMI (kg/m^2^), median (i.q.r.)	27.0 (24.0–30.0)	27.0 (24.0–27.0)	0.133	12
**Smoking status**				
Unknown	22 (4)	14 (6)	0.121	–
Smoker	89 (15)	31 (14)		
Non-smoker	390 (64.9)	144 (62.9)		
Former smoker	100 (16.6)	40 (18)		
**Co-morbidites**				
0	483 (80.4)	170 (74.2)	0.147	–
≥1	118 (19.6)	59 (26)		
**Medications**				
0	208 (34.6)	66 (29)	0.193	–
1	171 (28.5)	65 (29)		
2	141 (23.5)	52 (23)		
≥3	81 (14)	46 (20)		
**ASA grade**				
I	205 (34.1)	69 (30)	0.214	–
II	345 (57.4)	125 (54.6)		
III	51 (8)	35 (15)		

Values are *n* (%) unless otherwise indicated. BCS, breast-conserving surgery; RT, radiotherapy; SMD, standardized mean difference; i.q.r., interquartile range.

**Table 2:** Clinical and treatment characteristics for the BCS + RT and mastectomy groups

	BCS + RT (*n* = 601)	Mastectomy (*n* = 229)	SMD	Missing
**Tumour side**				
Right	289 (48.1)	107 (46.7)	0.027	–
Left	312 (51.9)	122 (53.3)		
**cT category**				
cT1	468 (77.9)	118 (51.5)	0.573	–
cT2	133 (22.1)	111 (48.5)		
**cN category**				
cN0	570 (94.8)	193 (84.3)	0.351	–
cN1	31 (5)	36 (16)		
**Invasive tumours**				
Unifocal	517 (86.0)	127 (55.5)	0.713	–
Multifocal	84 (14)	102 (44.5)		
**Largest invasive tumour size (mm), median (i.q.r.)**	15.0 (12.0–19.0)	19.0 (15.0–25.0)	0.638	–
**pN category**				
pN0	455 (76.2)	138 (60.5)	0.376	5
pN1–3	126 (21.1)	70 (31)		
≥pN4	16 (3)	20 (9)		
**Histological grade**				
1	120 (20.0)	26 (12)	0.255	3
2	329 (54.8)	128 (56.4)		
3	151 (25.2)	73 (32)		
**ER status**				
Negative	53 (9)	27 (13)	0.102	54
Positive	509 (90.6)	187 (87.4)		
**PR status**				
Negative	123 (21.9)	54 (25)	0.078	55
Positive	438 (78.1)	160 (74.8)		
**HER2 status**				
Negative	534 (89.1)	195 (85.2)	0.120	2
Positive	65 (11)	34 (15)		
Triple-negative breast cancer*	36 (6)	12 (5)	0.033	55
**Adjuvant treatment**				
Radiotherapy	595 (98.8)	92 (40)	1.636	8
Chemotherapy	217 (36.3)	99 (44)	0.166	9
Endocrine therapy	525 (87.5)	201 (90.1)	0.084	7
Anti-HER2 therapy	55 (9)	29 (13)	0.122	8

Values are *n* (%) unless otherwise indicated. *Variable excluded from matching/weighting/adjustment due to high level of missing data. BCS, breast-conserving surgery; RT, radiotherapy; SMD, standardized mean difference; i.q.r., interquartile range; ER, oestrogen receptor; PR, progesterone receptor; HER2 human epidermal growth factor receptor 2.

### Statistical analysis

Demographic and clinical characteristics were evaluated using descriptive methods. Categorical variables are summarized as *n* (%) and quantitative variables are summarized as median (interquartile range (i.q.r.)) for each treatment group (BCS + RT and mastectomy).

Furthermore, three different strategies were applied to balance the treatment groups. First, a 1 : 1 PSM strategy was implemented, followed by a 2 : 1 PSM strategy. In addition, an inverse probability of treatment weighting (IPTW) approach was used, applying weights based on the average treatment effect (ATE). These methods were used to reduce confounding and improve comparability between patients who underwent BCS + RT and patients who underwent mastectomy.

The most important covariates, pre-specified in the analysis, were selected for inclusion in the propensity score models. These covariates were subsequently evaluated for balance between the treatment groups and the results are presented as standardized mean differences (SMDs) in balancing plots for each matching/weighting strategy.

Time-to-event analyses were conducted to evaluate survival outcomes. OS was defined as the time from the index date to death from any cause. Patients who were alive at the end of follow-up were censored at the last date they were known to be alive. BCSS was defined as the time from the index date to death attributable to breast cancer. Deaths from other causes were censored at the time they occurred.

Kaplan–Meier curves are used to present the time-to-event outcomes and differences between treatment groups were compared using the log rank test. Finally, Cox proportional hazards regression analyses were performed, yielding HR (95% c.i.) values. Univariable Cox models were fitted for each of the three matched/weighted cohorts (1 : 1 PSM, 2 : 1 PSM, and IPTW ATE). In addition, a multivariable Cox model was performed in the full cohort, adjusting for all covariates included in the balancing procedures.

A statistical significance level of 5% was applied throughout all analyses. All statistical analyses were conducted using R, version 3.4.1 (R Foundation for Statistical Computing, Vienna, Austria). The CAN-study was approved by the Regional Ethical Committee in Uppsala (Dnr 2013/314), with an amendment approved by the Swedish Ethical Review Authority for this analysis (Dnr 2024-01530-02).

## Results

Out of 1670 patients in the randomized CAN-study, the final cohort used in the present analysis included 830 women with T1–2 invasive breast cancer. Patients from China were not included. In addition, 9 men, 379 patients with tumours >30 mm, <10 mm, or of unknown size, 32 women who received neoadjuvant treatment, and 54 women who underwent BCS without RT were excluded (*Fig. 1*).

### Characteristics of the study population

Among the 830 women, 601 underwent BCS + RT and 229 underwent mastectomy. The median age was 64 (i.q.r. 53–70) years for the BCS + RT group and 68 (i.q.r. 53–76) years for the mastectomy group (*Table 1*). In the mastectomy group, more women had co-morbidities, according to the Charlson Co-morbidity Index (CCI), and severe systemic disease, according to the ASA Physical Status Classification System (*Table 1*).

Tumour characteristics for the two treatment groups are shown in *Table 2*. The tumours in the mastectomy group were generally larger and had more lymph node involvement. In addition, a higher proportion of women in the mastectomy group had multifocal tumours compared with women in the BCS + RT group (44.5% *versus* 14%). Furthermore, a higher proportion of the tumours in the mastectomy group were grade 3, ER negative, and HER2 positive. In terms of adjuvant therapy, women who underwent mastectomy were more likely to receive chemotherapy (44% *versus* 36.3%).

### Survival

**Fig. 2:**
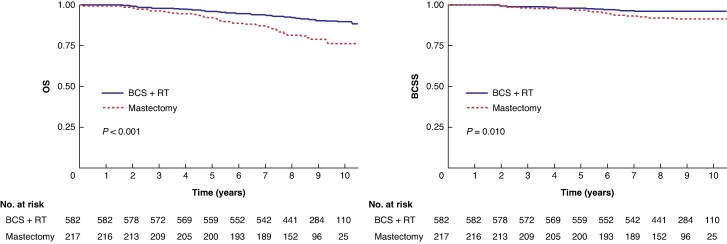
OS and BCSS for BCS + RT *versus* mastectomy OS, overall survival; BCSS, breast cancer-specific survival; BCS, breast-conserving surgery; RT, radiotherapy.

After 10 years of follow-up, the OS was 89.7% (95% c.i. 87.0% to 92.5%) in the BCS + RT group and 76.4% (95% c.i. 70.3% to 83.0%) in the mastectomy group. The corresponding BCSS was 96.3% (95% c.i. 94.8% to 97.9%) and 91.5% (95% c.i. 87.6% to 95.5%) respectively. In the unadjusted crude analysis of the full cohort, women who underwent mastectomy had significantly worse OS and worse BCSS compared with women who underwent BCS + RT (log rank test *P* < 0.001 and *P* = 0.010 respectively) (*Fig. 2*). In multivariable Cox regression analyses, mastectomy was again associated with a statistically significant less favourable outcome regarding OS in both the unadjusted and adjusted analyses, with an unadjusted HR of 2.45 (95% c.i. 1.65 to 3.63) and an adjusted HR of 1.70 (95% c.i. 1.05 to 2.76). BCSS was statistically significantly worse in the mastectomy group in the unadjusted analysis, with an HR of 2.27 (95% c.i. 1.20 to 4.30), whereas, in the adjusted analysis, BCSS was equal in the treatment groups, with an adjusted HR of 1.02 (95% c.i. 0.43 to 2.24) (*Table 3*).

**Table 3:** HR (95% c.i.) values estimated using a Cox proportional hazards regression model for the different populations of interest

Mastectomy *versus* BCS + RT	Overall mortality		Breast cancer-specific mortality	
	HR (95% c.i.)	*P*	HR (95% c.i.)	*P*
Unadjusted model	2.45 (1.65,3.63)	0.000	2.27 (1.20,4.30)	0.012
Adjusted model*	1.70 (1.05,2.76)	0.030	1.02 (0.43,2.24)	0.959
1 : 1 PSM model	1.52 (0.89,2.58)	0.123	1.09 (0.48,2.48)	0.830
2 : 1 PSM model	1.78 (1.09,2.91)	0.021	1.42 (0.65,3.11)	0.381
IPTW ATE model	1.85 (1.14,3.01)	0.013	1.21 (0.57,2.57)	0.628

^*^Adjusting for all covariates included in the balancing procedures. BCS, breast-conserving surgery; RT, radiotherapy; PSM, propensity score matched; IPTW, inverse probability of treatment weighting; ATE, average treatment effect.

**Fig. 3:**
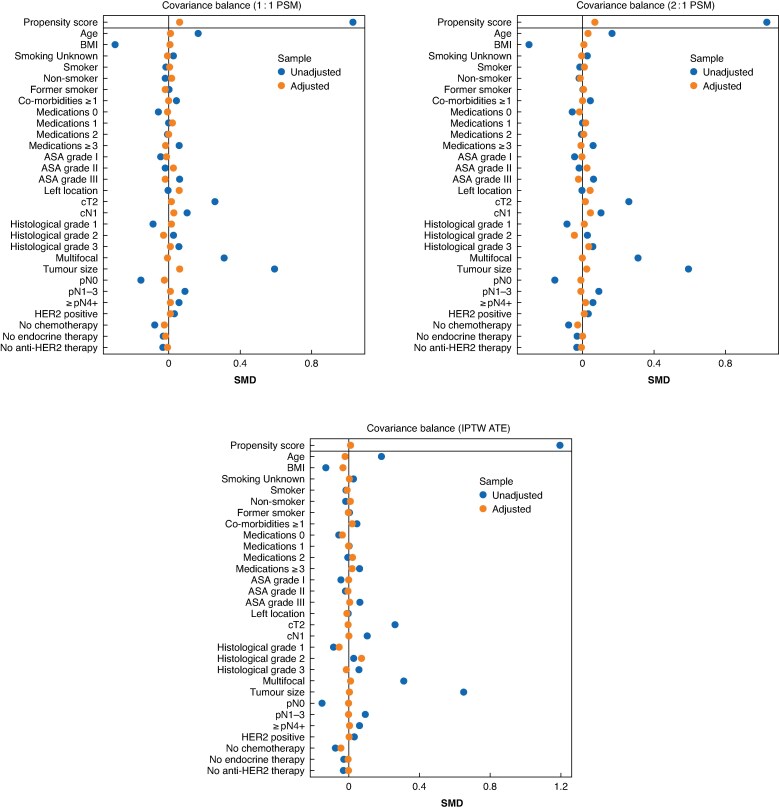
Balancing plots with SMDs for all baseline covariates before and after adjustment using the 1 : 1 PSM, 2 : 1 PSM, and IPTW ATE models Values closer to zero indicate better covariate balance. SMDs, standardized mean differences; PSM, propensity score matched; IPTW, inverse probability of treatment weighting; ATE, average treatment effect; HER2, human epidermal growth factor receptor 2.

Balancing plots with SMDs for all baseline covariates before and after adjustment using the 1 : 1 PSM, 2 : 1 PSM, and IPTW ATE models are presented in *Fig. 3*. In the first matched cohort (1 : 1 PSM), 178 well-balanced pairs were included in the analysis. In the second matched cohort (2 : 1 PSM), a total of 270 women who underwent BCS + RT and 178 women who underwent mastectomy were included. In the weighted analysis using IPTW based on the ATE, the effective sample comprised 587 BCS + RT patients and 210 mastectomy patients.

**Fig. 4:**
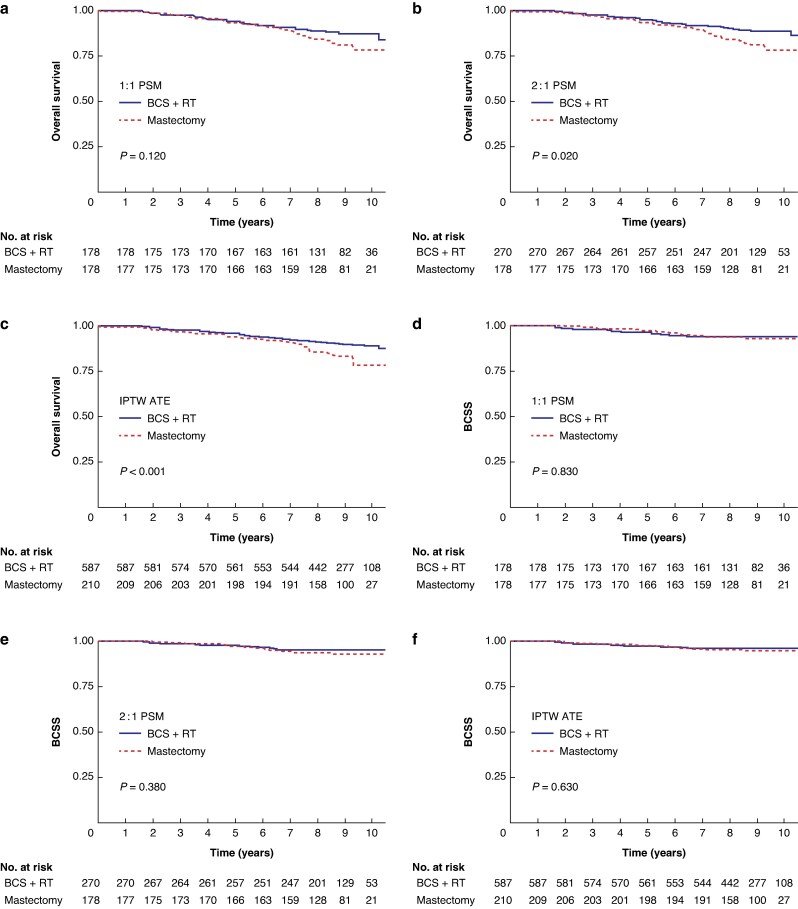
Survival **a–c** OS for BCS + RT *versus* mastectomy in the three different matched/weighted cohorts. **d–f** BCSS for BCS + RT *versus* mastectomy in the three different matched/weighted cohorts. OS, overall survival; BCS, breast-conserving surgery; RT, radiotherapy; BCSS, breast cancer-specific survival; PSM, propensity score matched; IPTW, inverse probability of treatment weighting; ATE, average treatment effect.

The Kaplan–Meier OS curves for the three different propensity score models are shown in *Fig. 4a–c*. There was statistically significant better OS after BCS + RT in the 2 : 1 PSM cohort, and in the IPTW ATE cohort, but no statistically significant difference in the 1 : 1 PSM cohort. The curves started to separate 2–6 years after surgery in the different cohorts. Finally, the analyses of BCSS did not show any statistically significant differences between BCS + RT and mastectomy using the 1 : 1 PSM (*P* = 0.830), 2 : 1 PSM (*P* = 0.380), or IPTW ATE (*P* = 0.630) propensity score model (*Fig. 4d–f*). A sensitivity analysis, excluding patients with HER2-positive and triple-negative breast cancer, showed similar results (*Table S1*).

The registered causes of death were analysed and showed that patients who underwent mastectomy, even after matching, more often died due to non-cancerous causes than patients who underwent BCS + RT (*Table S2*).

## Discussion

In this comparison of the prognosis of patients with breast cancer who underwent BCS + RT or mastectomy within a randomized anaesthesiology trial, there were no differences in BCSS when patients were balanced using three different propensity score models. OS was better after BCS + RT using two of the three propensity score models, but the curves started to separate after several years, indicating that it was not the type of surgery by itself affecting the prognosis. Moreover, women in the mastectomy group more often died due to non-cancerous causes.

The aim of this analysis was to mimic a randomized study as closely as possible. Including patients from an RCT helped to exclude patients who were not fit to understand study information or sign an informed consent form. To the authors’ knowledge, this approach has not been used before in contemporary register and observational studies. In the adjusted analyses and in the propensity score analyses, postoperative tumour data were used, which could possibly have affected the results compared with a randomized study, where the inclusion would depend on preoperative tumour biology and imaging data. On the other hand, trying to emulate a randomized study, the inclusion criteria were chosen with the purpose of excluding patients with occult reasons (for example patient or surgeon preference) for choosing one surgical treatment method over the other. Women with very small tumours, where BCS would have been the natural choice, were excluded, as were women with tumours >30 mm, even if BCS might have been possible for such women.

It has been suggested that the superior outcomes observed with BCS + RT in contemporary register and observational studies, as opposed to the findings from earlier randomized trials showing equal outcomes, may be attributable to advances in treatment, including more precise margin assessment and improved RT techniques. Advances in treatment have led to a decrease in locoregional recurrences, which, in turn, has improved survival^22^. This trend is considered to have had a more favourable impact on outcomes after BCS compared with mastectomy^23^. RT has been suggested to exert some protective effect on axillary recurrence rates by controlling any minimal residual disease and it has been suggested that the abscopal effect of RT may confer a survival advantage^24,25^. An alternative explanation for the better outcomes after BCS + RT compared with the results from earlier randomized studies could be that the introduction of mammographic screening has resulted in tumours being significantly smaller and more indolent. These types of tumours were probably not included in the studies from the 1980s and 1990s. For a subset of patients with small tumours selected for BCS today, it is conceivable that adjuvant RT (or even surgery) is overtreatment^26^.

In the randomized studies by Veronesi *et al*.^1^ and Fisher *et al*.^27^, 701 patients (two treatment arms) and 1843 patients (three treatment arms) were included. After 5 years of follow-up, there was a statistically significant OS and disease-free survival advantage for BCS + RT in the study by Fisher *et al*.^27^, but no differences after longer follow-up^2,4^. Moreover, there were no differences in the study by Veronesi *et al*.^1^ or after 20-year follow-up^3^. These are the main two studies that new data must be compared with and it is unlikely that a new randomized study will be performed, as it is already known that BCS + RT is equally good as mastectomy. Notwithstanding, a power calculation for a fictive randomized study comparing mastectomy and BCS + RT based on the assumption of the same survival as of the patients in the CAN-study was performed and, to be able to detect an OS benefit of 3%, the study would need 876 patients, which is about the same number as in this study mimicking a randomized study. Therefore, although a large number of patients from the initial CAN-study were lost due to exclusion and only approximately one-third of the remaining patients underwent mastectomy, the total number of patients included in the present study (that is 830 patients) is considered sufficient to answer the research question, while reducing selection bias to a minimum. Observational studies will include the same risk and type of selection bias regardless of the number of patients included.

Women who underwent mastectomy were unhealthier and their tumour biology was more aggressive than women who underwent BCS + RT. After adjusting for patient characteristics and tumour biology, there was no statistically significant difference regarding BCSS (HR 1.02 (95% c.i. 0.43 to 2.42)), while still a statistically significant difference for OS (HR 1.70 (95% c.i. 1.05 to 2.76)). After propensity score matching/weighting there were no differences in BCSS up to 10 years after surgery. For OS, there was a better outcome for BCS + RT and the differences were statistically significant in two of the three propensity score analyses. However, the survival curves started to separate several years after surgery in all models. This makes it unlikely that an increased risk of morbidity—and consequently mortality—associated with mastectomy explains the better OS observed after BCS + RT. Instead, it is hypothesized that some remaining selection bias exists that cannot be clearly identified. Looking at the causes of death in this study gives some indications in that direction, as mastectomy patients more often died due to non-cancerous causes, even after matching.

Interestingly, the OS curves in two contemporary non-randomized studies, with several thousands of patients included, started to separate immediately after surgery^14,28^. In the present study, the OS curves started to separate after 2 years in the crude analysis and after 2–6 years in the matched/weighted analyses. The risk of selection bias is supported by a Danish study, where patients who were initially assigned to BCS, but ended up being treated with mastectomy, had significantly better survival than patients for whom mastectomy was decided upfront^29^. This underlines a key limitation of observational studies, as they typically rely on matching and/or adjustment for postoperative pathological stage (pTNM), whereas treatment decisions are based on preoperative clinical and radiological staging and individual patient factors.

Consistent with previous studies, the present data demonstrate that patients undergoing mastectomy tend to have a greater co-morbidity burden and more unfavourable tumour characteristics^7–9,11–15,18,23,28^. Three propensity score models were used to compare well-balanced patient populations from within a randomized clinical study and no differences in BCSS were seen. There was an OS benefit for BCS + RT in crude, adjusted, and two of the three propensity score analyses, but the analyses indicate that this is due to occult selection bias.

## Supplementary Material

znag036_Supplementary_Data

## Data Availability

Anonymized individual patient data collected for the CAN-study can be made available on request via Mats Enlund, Centre for Clinical Research, Uppsala University, Västmanland Hospital, Västerås, 721 89, Sweden, considering possible legal restrictions (Swedish and European Union; General Data Protection Regulation) and after presenting a sound proposal and ethics approval. A signed data access agreement is mandatory, including a description of the conditions for data release and the requirements for data transfer, storage, archiving, publication, and co-authorship. Also, a study protocol, a statistical analysis plan, and an informed consent form may be asked for.
