# Supplementary material for: Survival after breast-conserving surgery and radiotherapy versus mastectomy: propensity score analyses within a randomized anaesthesiology trial
Source: Br J Surg. 2026 Apr 7;113(4):znag036. doi: 10.1093/bjs/znag036 (PMC13112022; doi:10.1093/bjs/znag036)
Supplement: znag036_Supplementary_Data [file znag036_supplementary_data.docx]

**Title: Survival after breast-conserving surgery and radiotherapy versus mastectomy: a propensity score-matched analysis within a randomized clinical anaesthesiologic study**

Authors Charlotta Wadsten^1,2^, Anders Berglund^3^, Emma Söderberg^1,2^, Greger Nilsson^4,5,6^, Leif Bergkvist^7^, Mats Enlund^7,8,9^, and Fredrik Wärnberg^10,11^

^1^ Department of Surgery, Sundsvall Hospital, Sundsvall, Sweden

^2^ Department of Diagnostics and Intervention, Surgery, Umeå University, Umeå, Sweden

^3^ Department of Statistics, Epistat AB, Uppsala, Sweden

^4^ Department of Immunology, Genetics and Pathology, Uppsala University, Uppsala, Sweden

^5^ Department of Oncology, Gävle Hospital, Gävle, Sweden

^6^ Department of Oncology, Visby Hospital, Visby, Sweden

^7^ Centre for Clinical Research, Uppsala University and Region Vastmanland, Vastmanland Hospital, Västerås, Sweden

^8^ Department of Surgical Sciences, Division of Anesthesiology and Intensive Care, Uppsala University, Uppsala, Sweden

^9^ ESAIC Onco Anesthesiology Research Group, EuroPeriscope

^10^ Department of Surgery, Sahlgrenska University Hospital, Gothenburg, Region Västra Götaland, Sweden

^11^ Institution of Clinical Sciences, Sahlgrenska Academy at Gothenburg University, Gothenburg, Sweden

**Corresponding author.** Charlotta Wadsten, Dept of Surgery, Sundsvall Hospital, 851 86 Sundsvall, Sweden **ORCID ID 0000-0003-0571-7265**; **Twitter** @WadstenLotta

**Supplementary Figures and Tables**

**Supplementary Table 1.** Hazard ratios (HRs) and 95% confidence intervals (CIs) estimated using a Cox proportional hazards regression modes for the different populations of interest restricted to patients without HER2-positive or triple negative breast cancer

|  |  |  |  |  |  |  |  |  |  |
| --- | --- | --- | --- | --- | --- | --- | --- | --- | --- |
|  | **Overall mortality** | | | |  | **Cause-specific mortality** | | | |
| **Mastectomy vs BCS + RT** | **HR** | **95% CI** | | **P-value** |  | **HR** | **95% CI** | | **P-value** |
| Adjusted model* | 1.84 | 1.11 | 3.06 | 0.018 |  | 1.22 | 0.47 | 3.16 | 0.675 |
| PSM matched cohorts (ratio 1:1) | 2.12 | 1.18 | 3.79 | 0.012 |  | 1.47 | 0.56 | 3.86 | 0.435 |
| PSM matched cohorts (ratio 2:1) | 1.96 | 1.19 | 3.23 | 0.009 |  | 1.44 | 0.61 | 3.39 | 0.406 |
| IPTW model | 2.09 | 1.22 | 3.59 | 0.007 |  | 1.41 | 0.64 | 3.12 | 0.397 |
|  |  |  |  |  |  |  |  |  |  |

* adjusting for all covariates included in the balancing procedures.

BCS=breast conserving surgery, RT=radiotherapy, HR=hazard ratio, CI=confidence interval, PSM=propensity score matched, IPTW= inverse probability of treatment weighting

**Supplementary Table 2.** Causes of death by treatments in the propensity score matched cohort (ratio 1:1)

|  |  |  |
| --- | --- | --- |
| **Cause of death** | **BCS + RT** | **Mastectomy** |
| Breast cancer | 7 | 8 |
| Cancer, other | 3 | 2 |
| Other causes | 9 | 18 |

BCS=breast conserving surgery, RT=radiotherapy
